# 870 Cannabis Intoxication Does Not Impact Nutritional Status in Patients with Small Burns

**DOI:** 10.1093/jbcr/iraf019.401

**Published:** 2025-04-01

**Authors:** Sarah Wang, Amara Emeh, Eloise Stanton, Sunnie Wong, Artur Manasyan, Elizabeth Boudiab, Deborah Choe, Maxwell Johnson, Haig Yenikomshian, Justin Gillenwater

**Affiliations:** University of California Keck School of Medicine; University of California Keck School of Medicine; University of Southern California; Los Angeles General Medical Center; University of California Keck School of Medicine; Los Angeles General Medical Center; University of California Keck School of Medicine; University of California Keck School of Medicine; University of California Keck School of Medicine; Los Angeles General Medical Center

## Abstract

**Introduction:**

Burn patients exhibit one of the most intense hypermetabolic responses among critically ill populations, making them highly susceptible to malnutrition—linked to prolonged hospital stays and delayed wound healing. While cannabis is recognized for its appetite-stimulating properties in acute settings, its interaction with the unique metabolic demands of burn injuries remains underexplored. We hypothesize that cannabis use may positively influence pre-injury nutritional status, thereby improving clinical outcomes in burn patients.

**Methods:**

A single-institution retrospective study was conducted on burn patients who tested positive for cannabis intoxication between 2015 and 2024. Eligible patients included those with burns < 20% total body surface area (TBSA) and a positive cannabis result on admission urine toxicology. These patients were matched 1:1 with controls—based on age, gender, TBSA, and length of stay (LOS)—who tested negative for cannabis. The primary predictor variable was cannabis use, while primary outcomes included burn parameters, prealbumin and albumin levels, overall outcomes, and complications. Statistical analyses using Stata included Student’s t-test, chi-square, and multiple regression, with significance set at p< 0.05.

**Results:**

We analyzed 76 cannabis positive burn patients and 76 controls. No significant differences were found in race (p= 0.494), ethnicity (p= 0.598), alcohol use (p= 0.087), number of complications (p= 0.092), number of OR visits (p= 0.909), discharge disposition (p= 0.382), or mortality (p= 0.083). There was a significant relationship between inhalation injury and cannabis use (p= 0.023), with fewer cannabis positive patients experiencing inhalation injury (0% vs 6.58%). When controlling for BMI, multiple regression analysis indicated that cannabis intoxication was not significantly associated with changes in admission prealbumin (18.8 vs 19.2, p= 0.804) or admission albumin (3.9 vs 4.0, p= 0.375) levels. There was also no significant variation in the number of days post-admission required to achieve peak prealbumin (3.8 days vs 3.9 days, p= 0.876) and albumin level (0.3 days vs 1.0 days, p= 0.088). Increased age was associated with a reduction in admission albumin (p< 0.001), and Caucasian patients had increased albumin compared to other races (p= 0.048).

**Conclusions:**

Contrary to our hypothesis, cannabis intoxication had no significant association with nutritional status. While cannabis use was not associated with mortality, it was associated with a decrease in inhalation injury. Further research with larger sample sizes is necessary to fully understand the complex interactions between cannabis use and burn outcomes.

**Applicability of Research to Practice:**

This study emphasizes the need for further research to explore how cannabis use may impact other clinical outcomes to inform comprehensive burn care.

**Funding for the Study:**

N/A

